# The successful search for genetic loci associated with depression

**DOI:** 10.1186/s13073-015-0217-4

**Published:** 2015-08-25

**Authors:** Margarita Rivera, Peter McGuffin

**Affiliations:** Instituto de Investigación Biosanitaria ibs, Granada and CIBER en Salud Mental (CIBERSAM), University of Granada, Granada, Spain; MRC Social Genetic and Developmental Psychiatry Centre, Institute of Psychiatry, Psychology & Neuroscience, King’s College London, London, SE5 8AF UK

## Abstract

Major depressive disorder is among the leading causes of disease burden and disability, as well as a major public health concern worldwide. Despite its substantial heritability, no robustly replicated genetic risk loci had been found until recently. Now, a new study has identified, and replicated, two variants associated with an increased risk for this disorder. The success of this study appears to lie in the use of low-coverage sequencing, instead of microarrays, and in minimizing phenotypic and genetic heterogeneity.

## The pursuit of a single genetic cause

Major depressive disorder (MDD) is one of the most common mental illnesses and the second-leading cause of disability worldwide, making it the most frequent cause of absenteeism from work and a major contributor to the burden on healthcare systems [1]. Numerous attempts had been made to pin down a single simple genetic cause, but no significant associations had been found. Instead, the recent focus had begun to shift to viewing depression as a complex disease, with an increasing emphasis on the interplay between multiple genes and environmental factors. However, in an article published recently in *Nature*, the China, Oxford and Virginia Commonwealth University Experimental Research on Genetic Epidemiology (CONVERGE) consortium has identified, and replicated in an independent sample, two variants associated with an increase in risk of MDD [2].

There are several potential explanations both for why previous studies failed to identify loci that are significantly associated with depression and for the success of the CONVERGE group. One key factor seems to lie in the careful phenotyping for more-severe forms of depression and the choice of low-coverage sequencing by CONVERGE. Here, we discuss these factors and their implications for future studies.

## The genetic basis of depression

Family, twin and, to a lesser extent, adoption studies provide compelling evidence that genetic factors are involved in susceptibility to MDD [3]. Heritability estimates range from 17–75 %, with a mean of 37 %, and there is evidence that severity and recurrence are associated with higher heritability [3]. Until comparatively recently, genetic association studies have focused mainly on candidate genes — for example, those involved in neurotransmission or in the hypothalamic–pituitary–adrenal (HPA) axis. Almost 200 genes have been investigated with this approach, although only a few findings have been replicated and yielded significant results [4]. As with other common familial disorders, the emphasis subsequently shifted to genome-wide association studies (GWAS). These have been less successful so far in finding robust evidence for genetic loci that contribute to susceptibility to MDD than has been the case in other psychiatric disorders such as schizophrenia and bipolar disorder [5]. A recent mega-analysis conducted on eight GWAS, including 9240 MDD cases and 9519 controls, analyzed more than 1.2 million single-nucleotide polymorphisms (SNPs) in the discovery phase but yielded no convincing results of genome-wide significance (at *P* < 5 × 10^−8^) [5].

There are several plausible explanations for this failure. The first is that diagnostic and/or etiological heterogeneity has been a barrier to detection. For example, milder forms of depression might not be influenced by the same genetic risk factors as more-severe forms of the disorder, and environmental factors could be more important in some cases of depression compared with others. Another factor is that the common variants contributing to depression might have very small genetic effects, and so even-larger sample sizes are required to detect any associations. In addition, many genome-wide searches have been insufficiently sensitive to detect rare types of structural variation with possibly larger effects, such as copy number alterations (CNAs). Failure to take into account environmental factors that could act in combination with genetic risk factors might also explain the failure to detect the latter, and methodological factors, such as insufficient quality control in combining samples and multiple testing issues, might have stood in the way [6, 7].

The COVERGE consortium was able to overcome these limitations and was able to identify new associations. It recruited 5303 Han Chinese women with recurrent MDD and 5337 healthy control individuals from 58 Chinese hospitals. All the participants were genotyped using a low-coverage sequencing method. The results showed two loci significantly associated with MDD at a genome-wide level on chromosome 10, one near the *SIRT1* gene (rs12415800; *P* = 1.92 × 10^−8^) and the other in an intron of the *LHPP* gene (rs35936514; *P* = 1.27 × 10^−8^). To confirm this finding, the authors used a different method to re-genotype these individuals, and the results supported the association of these two SNPs with MDD [2]. These associations were also replicated in an independent cohort of 3231 Han Chinese women with recurrent MDD and 3186 controls, confirming that the SNPs at *SIRT1* and *LHPP* genes are significantly associated with MDD [2].

The authors then focused on cases within the CONVERGE consortium diagnosed with melancholia, a more severe and possibly more heritable subtype of MDD. The results showed genome-wide significant associations with the same two loci on chromosome 10. Interestingly, the association at the *SIRT1* gene was more significant than when MDD as a whole was studied (*P* = 2.95 × 10^−10^) [2]. The *SIRT1* gene, which encodes an NAD-dependent protein deacetylase, is known to be involved in mitochondrial metabolism [8], and intriguingly the same group has reported recently that MDD is associated with an increased amount of mitochondrial DNA [9].

Unfortunately, the CONVERGE group failed to replicate their top signals in the Psychiatric Genomics Consortium (PGC) mega-analysis of European cohorts [5]. Furthermore, the PGC found no evidence implicating mitochondrial metabolism when they studied over 60,000 participants and performed pathway analysis in MDD and other major psychiatric disorders [10]. One important factor to be taken into consideration is that the variants found associated with MDD in Chinese individuals are less common in European populations.

## Why did the CONVERGE study succeed?

One might argue that a key question concerns why the CONVERGE study succeeded when the PGC and others so far have failed. The CONVERGE study attempted to minimize phenotypic and genetic heterogeneity by choosing women only and concentrating on more-severe hospital-treated cases of MDD from China. The focus was thus on a less prevalent type of depression than that included in the PGC from Europe and the USA, which included population-ascertained as well as clinically ascertained samples. The participants in the CONVERGE study were genotyped using a low-coverage sequencing approach rather than a microarray-based GWAS. This is the first study published, to date, using this approach to investigate the genetic contribution to MDD. The authors of the CONVERGE study argue that the method allowed them to capture a greater repertoire of variants than can be detected by commercially available SNP-based genotyping arrays.

## Concluding remarks

The results of the CONVERGE study are provocative, but it is important to consider what needs to be done next to clarify the molecular-genetic contribution to MDD. MDD is a much more common disorder (or group of disorders) than schizophrenia or bipolar disorder, in which the PGC approach of combining samples from multiple centres has been successful. Perhaps, therefore, larger-scale ascertainment of more-homogeneous, more severely affected samples is the key to enhancing the chances of success in MDD. Further replications in very large independent cohorts, as well as carefully matched case–control samples from a different ethnic origin, would be desirable. It would also be of interest to extend genomic analysis of MDD to new approaches such as gene–gene and gene–environment interactions and pathway analysis. Thus, the intriguing CONVERGE study findings open the door to a range of further studies that could help to identify biological pathways and provide therapeutic targets for MDD, a common illness with major impact on public health and on the lives of many people.

## Abbreviations

CNA: Copy number alteration
GWAS: Genome-wide association study
HPA: Hypothalamic–pituitary–adrenal
MDD: Major depressive disorder
PGC: Psychiatric Genomics Consortium
SNP: Single-nucleotide polymorphism
